# Editorial

**Published:** 1847-06

**Authors:** 


### THE MEDICAL EXAMINER.

PHILADELPHIA, JUNE, 1847.

### NATIONAL MEDICAL CONVENTION.

In our *Record* department of the present number of the Examiner, will be found the Minutes of the Proceedings of the Medical Convention, which assembled in this city on the 5th ultimo. To bring the account within the limits of a single number, we have been obliged to omit the names of the delegates, and to defer to future numbers the reports made by the several committees appointed at the previous Convention.

Among the resolutions reported by the committees was one recommending to the medical schools of the country to extend the lecture term to six months, and to demand of the medical student higher preliminary attainments. On another page we have indulged in some strictures on the proceedings in reference to the latter point, expressive of our surprise at the very slender, we might say, derogatory amount of literary knowledge, suggested as sufficient by gentlemen who have been so anxious to *elevate* the professional standard. In regard to an extension of the lecture term by the colleges, much may be said for and against the scheme; but one thing is most certain, that it cannot be accomplished without a pretty general concurrence among the colleges themselves, and present indications do not seem to warrant the belief that such will be the case. We infer this from past experience, but more particularly from the following remarks contained in the Boston Medical and Surgical Journal of the 19th ult., the editor of which was a member of the Convention, and is withal well acquainted with every thing pertaining to the New England Medical Schools, but not being in any manner connected with them, can have no bias other than that resulting from a more intimate acquaintance with the difficulties of the subject than is generally possessed, from having himself in former years been engaged in teaching

"One measure of the Convention, which proposes that the lecture terms shall be extended to six months in all the medical colleges in the Union, will be hard to digest, although carried triumphantly through the Convention. The fact is, these institutions are independent bodies, with chartered rights which cannot be wrenched from them. They may be induced by the force of public opinion to vary very considerably the present system of lecturing, but cannot be driven or persuaded to continue the annual series of lectures any longer than the judgment of the faculties determines. Students could not be kept together so long without some new and stronger motive than has yet been presented to them. Both professors and pupils, we fear, would loathe the sight of each other before the term was brought to a close. In the fickle climate of New England it is a question whether either party could endure the fatigue of a six months session, without suffering in health. Again, at least one-third, if not more, of the students entering upon the study of medicine, could not procure the means of defraying their personal expenses, six months in succession. But as the ground was thoroughly surveyed by the advocates and opponents of the scheme, we shall neither go over it again, nor present a category of new arguments in opposition to the expressed will of a majority, but frankly state what we believe to be true, viz., that New England will not conform to the proposed measure, and that the institution which first announces the long term system will droop and die.

The meeting of the Convention in Philadelphia was delightful, and our recollections are of a very pleasant kind. At no period in the history of the republic, have so many physicians been together from its different portions. Good must result from the interviews which gentlemen had with each other; and the warm friendships that were commenced on that agreeable occasion, will be promotive of kindness and fraternal feeling. The public doings, also, of the Convention will exert a beneficial influence, although the progress of reform must necessarily be slow and perhaps tedious."

In the concluding remarks of our Boston brother, every member of the profession in Philadelphia will heartily concur. Whatever may result from the proceedings of the Convention,—in the number and respectability of those who composed it, and the dignity which prevailed thoughout all its sittings, there was much to be proud of. We believe, that all its members from a distance were impressed with the brotherly feeling manifested towards them, as well as towards each other, by the physicians of Philadelphia; and we speak advisedly, when we say that the latter were delighted with the opportunity afforded them of making the acquaintance of their distinguished brethren thoughout our great and glorious country.

---

### SHIP FEVER.

The vast number of emigrants arriving at some of our sea-ports, particularly New York, has become a source of considerable anxiety to the inhabitants. Not merely from the rapid accumulation of persons beyond the immediate means of employment, which constitutes a serious tax upon the people, but from the destitution of the poor creatures, and the crowded state of the vessels in which they are brought, many of them pass at once into the public institutions, affected with what is called ship fever; and this in some instances has extended to the inmates, and even the officers of the institutions into which they are received.

For weeks past we have had accounts of this state of things in the city of New York, and now, by the public prints, we learn that the same is occurring in the city of Baltimore. By a letter dated Baltimore, May 21, 1847, published in the United States Gazette, the writer says: "What is termed the ship fever is now prevailing to a considerable extent among the emigrants recently arrived at our port. In several instances it has proved fatal, and is said to be contagious. Dr. Lawrence, one of the health officers, was taken with it, and is now quite ill."

---

### OBITUARY.

Died, in the city of New York, on the 29th of April last, in the sixtieth year of his age, John Revere, M. D., Professor of the Theory and Practice of Medicine in the Medical Department of the University of the City of New York.

Dr. Revere was born in the city of Boston, and was the son of Paul Revere, a distinguished patriot of the Revolution. After completing his education at Cambridge, Mass., he entered as student of Medicine in the office of Dr. James Jackson, of Boston, and after the requisite period of study proceeded to Europe, where he spent several years in the pursuit of knowledge, and graduated at the University of Edinburgh, under the distinguished Professors Home, Hope, Hamilton, Monro, &c. Having thus honourably finished his education, he returned to his native city and commenced the practice of his profession, but was soon compelled to relinquish it, and fly to a more genial clime, in consequence of frequent inflammations of the lungs and threatened phthisis. A trip to Richmond, Va., where he spent some time, so far improved his health, that he was induced again to attempt the practice of his profession, and with that view he settled in Baltimore, where he remained several years, and then proceeded again to Europe on business. Whilst in Europe, he was elected to the chair of the Theory and Practice of Medicine in the Jefferson Medical College of Philadelphia, the duties of which he continued to discharge with great fidelity and ability for several years, when, in the year 1841, he was induced to resign, in order to accept a chair in the University of New York.

We had the pleasure of being associated with him in the Jefferson Medical College, and a more zealous instructer in the important branch which he taught could not be met with. Although his health was then somewhat delicate, he rarely failed to be at his post; and on all occasions of importance participated in the deliberations of his colleagues,—ever anxious to advance the great interests of the profession to which he belonged, and of the institution with which he was more immediately connected.

Died,—In Richmond, Virginia, on the —— ultimo, Augustus S. Warner, M.D., Professor of Surgery in the Hampden Sidney College. Professor Warner, we are informed, was an intrepid and successful surgeon, and an able and eloquent lecturer.

Died,—Suddenly in this city, on the 9th ultimo, in the 51st year of his age, George McClellan, M. D., formerly Professor of Surgery in the Medical Department of Pennsylvania College, and previously in Jefferson Medical College. Dr. McClellan was eminent as a surgeon, and particularly distinguished as a bold and ingenious operator, and an ardent and eloquent lecturer. The College of Physicians, of which he was a member, was convened on the occasion of his death, when the following proceedings were had:

"Whereas, By the sudden demise of Doctor George McClellan, in the period of maturity of intellect, and in the enjoyment of extensive professional usefulness and reputation, this College, of which the deceased was a Fellow, and the community at large, have sustained a loss which will be long felt, as it is now deeply deplored. Therefore,

*Resolved*, That the College will attend the funeral of its deceased Fellow George McClellan, as a mark of their respect for his talents and his worth.

*Resolved,* That the College tender to the family of the deceased their sincere sympathy and condolence for the very great and irreparable bereavement they have sustained in the death of a most kind and affectionate husband and parent.

*Resolved,* That Dr. Samuel G. Morton be requested to prepare a biographical notice of our deceased Fellow, to be read before this College.

*Resolved,* That a copy of these resolutions be transmitted to the family of the deceased, and that they be published in the daily papers, and in the Professional Journals of the city.

*Resolved,* That the College adjourn to meet at nine o'clock to-morrow morning, to proceed in a body to attend the funeral of their deceased Fellow."

